# Proteomic profiling reveals the potential mechanisms and regulatory targets of sirtuin 4 in 1-methyl-4-phenyl-1,2,3,6-tetrahydropyridine-induced Parkinson’s mouse model

**DOI:** 10.3389/fnins.2022.1035444

**Published:** 2023-01-25

**Authors:** Huidan Weng, Wenjing Song, Kangyue Fu, Yunqian Guan, Guoen Cai, En Huang, Xiaochun Chen, Haiqiang Zou, Qinyong Ye

**Affiliations:** ^1^Department of Neurology, Fujian Medical University Union Hospital, Fuzhou, China; ^2^Fujian Key Laboratory of Molecular Neurology, Institute of Neuroscience, Fujian Medical University, Fuzhou, China; ^3^Cell Therapy Center, Xuanwu Hospital, Capital Medical University, Beijing, China; ^4^The School of Basic Medical Sciences, Fujian Key Laboratory of Brain Aging and Neurodegenerative Diseases, Fujian Medical University, Fuzhou, China; ^5^Department of Neurosurgery, General Hospital of Southern Theatre Command, PLA, Guangzhou, Guangdong, China

**Keywords:** Parkinson’s disease, SIRT4, quantitative proteomic analysis, bioinformatics, biomarkers, FABP4, PPAR signaling pathway

## Abstract

**Introduction:**

Parkinson’s disease (PD), as a common neurodegenerative disease, currently has no effective therapeutic approaches to delay or stop its progression. There is an urgent need to further define its pathogenesis and develop new therapeutic targets. An increasing number of studies have shown that members of the sirtuin (SIRT) family are differentially involved in neurodegenerative diseases, indicating their potential to serve as targets in therapeutic strategies. Mitochondrial SIRT4 possesses multiple enzymatic activities, such as deacetylase, ADP ribosyltransferase, lipoamidase, and deacylase activities, and exhibits different enzymatic activities and target substrates in different tissues and cells; thus, mitochondrial SIRT4 plays an integral role in regulating metabolism. However, the role and mechanism of SIRT4 in PD are not fully understood. This study aimed to investigate the potential mechanism and possible regulatory targets of SIRT4 in 1-methyl-4-phenyl-1,2,3,6-tetrahydropyridine (MPTP)-induced PD mice.

**Methods:**

The expression of the SIRT4 protein in the MPTP-induced PD mouse mice or key familial Parkinson disease protein 7 knockout (DJ-1 KO) rat was compared against the control group by western blot assay. Afterwards, quantitative proteomics and bioinformatics analyses were performed to identify altered proteins in the vitro model and reveal the possible functional role of SIRT4. The most promising molecular target of SIRT4 were screened and validated by viral transfection, western blot assay and reverse transcription quantitative PCR (RT-qPCR) assays.

**Results:**

The expression of the SIRT4 protein was found to be altered both in the MPTP-induced PD mouse mice and DJ-1KO rats. Following the viral transfection of SIRT4, a quantitative proteomics analysis identified 5,094 altered proteins in the vitro model, including 213 significantly upregulated proteins and 222 significantly downregulated proteins. The results from bioinformatics analyses indicated that SIRT4 mainly affected the ribosomal pathway, propionate metabolism pathway, peroxisome proliferator-activated receptor (PPAR) signaling pathway and peroxisome pathway in cells, and we screened 25 potential molecular targets. Finally, only fatty acid binding protein 4 (FABP4) in the PPAR signaling pathway was regulated by SIRT4 among the 25 molecules. Importantly, the alterations in FABP4 and PPARγ were verified in the MPTP-induced PD mouse model.

**Discussion:**

Our results indicated that FABP4 in the PPAR signaling pathway is the most promising molecular target of SIRT4 in an MPTP-induced mouse model and revealed the possible functional role of SIRT4. This study provides a reference for future drug development and mechanism research with SIRT4 as a target or biomarker.

## 1. Introduction

Neurological diseases are the leading cause of disability/paralysis worldwide; notably, among these diseases, Parkinson’s disease (PD) has the fastest rate of increase in incidence and an increasingly younger onset, and it is closely related to damage to substantia nigra dopaminergic neurons (Dorsey and Bloem, 2018). The current studies examining the pathogenesis of PD focus on the misfolding and aggregation of α-synuclein, mitochondrial dysfunction, impaired protein clearance, neuroinflammation, and oxidative stress, which interact and lead to cascading and irreversible cellular damage (Michel et al., 2016; Jankovic and Tan, 2020). However, current therapeutic approaches are unable to stop the progression of the underlying neurodegenerative disease. Furthermore, the lack of reliable and sensitive biomarkers for the disease limits the development of neuroprotective therapies (Poewe et al., 2017). Therefore, a better understanding of the pathophysiology of neurodegenerative diseases such as PD and the identification of new therapeutic targets are crucial.

The SIRT family consists of seven proteins (SIRT1-SIRT7) with distinct nicotinamide adenine dinucleotide (NAD^+^)-dependent deacetylase and ADP ribosyltransferase activities. These proteins interact with a variety of signaling proteins and transcription factors and exhibit different enzymatic activities, targets, and subcellular localizations, resulting in powerful epigenetic regulation and posttranslational modifications (Tang, 2017). SIRT is expressed at high levels in the brain and is involved in a variety of cellular processes, including cell survival, chromatin remodeling, energy metabolism, neuroprotection, and tumor development; it is also an important regulator of biological processes (Lavu et al., 2008; Rose et al., 2017; Xu et al., 2020). An increasing number of studies have confirmed that mitochondrial sirtuins (SIRT3-SIRT5) stabilize respiratory chain function, increase the activity of mitochondrial respiratory chain complexes, increase energy metabolism, promote autophagy, inhibit oxidative stress and inflammatory responses. Consequently, these proteins have become important targets in PD research (He et al., 2022). Furthermore, activators or inhibitors of SIRT proteins in PD models also exert neuroprotective effects (Gomes et al., 2019). For example, resveratrol, a SIRT1 activator, reduces cell death in animal models of neurotoxin-induced PD (Lofrumento et al., 2014); SIRT2 inhibitors rescue α-syn-mediated PD toxicity in cell models (Outeiro et al., 2007; Singh et al., 2019). SIRT3 interacts with α-synuclein to exert a protective effect in a mouse model of MPTP-induced PD (Liu et al., 2015; Park et al., 2020). In summary, SIRT family proteins may affect the development of PD through diverse mechanisms.

Notably, SIRT4-7, which are currently less studied, play a key role in brain health (Yeong et al., 2020). SIRT4 expression and activity are mainly associated with susceptibility to endocrine diseases, tumors, and neurodegenerative diseases (Betsinger and Cristea, 2019). Related studies suggested that SIRT4 is a major regulator of mitochondrial metabolism and is widely involved in energy metabolism in mammalian cells, such as inhibiting insulin secretion, regulating mitochondrial ATP homeostasis, and modulating redox reactions (Li and Zheng, 2018). In addition, it affects leucine metabolism (Csibi et al., 2013; Komlos et al., 2013; Anderson et al., 2017). SIRT4 also increases ADP or AMP levels by inhibiting glutamine metabolism and blocking the mTOR signaling pathway, thus exerting its tumor-suppressive effects in hepatocellular carcinoma (Wang et al., 2019). In the central nervous system, SIRT4 overexpression prevents the differentiation of radial glial cells into astrocytes (Komlos et al., 2013). The recovery of olfactory function is associated with increased expression of medullary SIRT1 and SIRT4 (Marin et al., 2019). In addition, a neurotoxin (erythropoietin) induces increases in SIRT4 expression (Shih et al., 2014). Most importantly, SIRT4 overexpression increases the expression levels of GLT-1 and glutamate dehydrogenase (GDH), which results in avoiding the uptake of excess glutamate while preventing the conversion of glutamine to glutamate by inhibiting glutamine synthetase, thereby maintaining the levels of glutamate in the brain (Csibi et al., 2013; Yalçın and Colak, 2020). Although mitochondrial SIRT4 exhibits some therapeutic potential, SIRT4 remains the least studied SIRT, and its role in neurodegenerative diseases such as PD is largely unknown. This lack of evidence may be related to its low *in vitro* enzyme activity and the identification of fewer acting substrates. Members of the SIRT family share some protein substrates and physiological activities (Haigis and Sinclair, 2010), and different isoforms may compete for the same substrates to alter their acetylation status and activity (Luo et al., 2017). This complexity necessitates a better understanding of the mechanism of action and potential targets of SIRT4.

Fatty acid binding proteins (FABPs) have a central role in coordinating lipid transport, metabolism, and reactions in different tissues and organs, and different members are expressed in a tissue-specific manner to optimize local fatty acid utilization (Frizzell et al., 2020). FABP4 is one of these isoforms (Li et al., 2020). Increased concentrations of B-FABP are associated with human brain tumors (e.g., glioblastoma and astrocytoma), neurodegenerative diseases (Alzheimer’s disease and PD), and other cognitive dysfunctions (Choromańska et al., 2011). A series of studies suggest that FABP3, FABP5, and FABP7 play important roles in α-synuclein oligomerization and migration, trigger the loss of neuronal mitochondrial function, and even interact with VDAC-1 to modulate the formation of channel pores in the mitochondrial membrane (Kawahata et al., 2019; Matsuo et al., 2019; Cheng et al., 2021; Kawahata and Fukunaga, 2022). Thus, FABP is considered a promising therapeutic target for α-synucleinopathies (Kawahata and Fukunaga, 2022). Although FABP4 has been less studied in PD, previous studies suggest that FABP4 is a powerful and novel biomarker for acute stroke (Bacigaluppi and Martino, 2020), an effective target for diabetes treatment (Cao et al., 2013), a predictor of adverse cardiovascular events (Peeters et al., 2011), a key determinant of the metastatic potential of ovarian cancer (Gharpure et al., 2018) and a regulator of neuroinflammation and cognitive decline in obese mice (So et al., 2022), which indicates that further studies of SIRT4 in the field of PD are warranted. In human hepatocellular carcinoma cells (HepG2/HuH7), SIRT1-FOXO1 signaling promotes the increased secretion of FABP4 protein and neutral lipid accumulation (Attal et al., 2022). Although this study was not conducted using PD models, it also reveals the relevance of SIRT to FABP4.

Peroxisome proliferator-activated receptor (PPAR) is a key sensor and major regulator of cellular metabolism and a potential therapeutic target for Alzheimer’s disease, PD, Huntington’s disease and other degenerative diseases (Zolezzi et al., 2017). A significant reduction in the levels of PPARγ and FABP4 transcripts (key genes in the adipose network) has been observed in bone marrow stem cells from patients with atypical PD (Angelova et al., 2018). Based on this finding, FABP4 in the PPAR signaling pathway may play a role in PD-related disorders. In addition, a close link between the SIRT family and PPAR signaling has been identified. For example, SIRT binds to PPARα and protects the heart from hypertrophy, metabolic dysregulation, and inflammation (Planavila et al., 2011). SIRT6 promotes hepatic β-oxidation by activating PPARα (Naiman et al., 2019). In conclusion, the SIRT family, FABP4, and PPAR are more or less inextricably linked.

**FIGURE 1:**
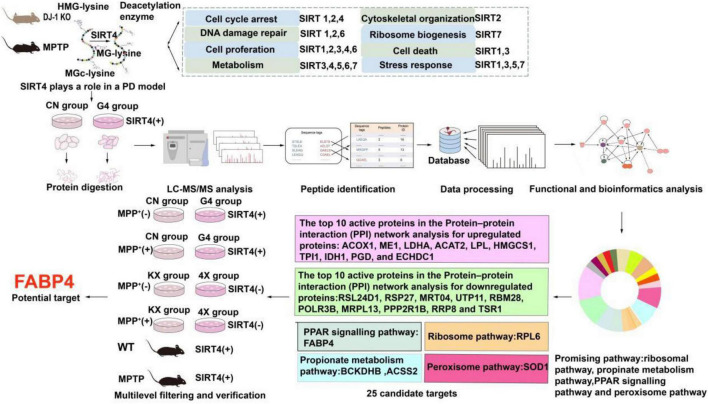
Workflow of the study. The summary of the main functions of SIRT family proteins come from reference (Imai and Guarente, 2014; Li et al., 2018).

Tandem mass tag (TMT)-labeled quantitative proteomics and liquid chromatography-tandem mass spectrometry (LC–MS/MS) have been used to analyse various biological processes and screen for promising molecular targets, which are useful for understanding molecular biological mechanisms (Moulder et al., 2018). As shown in Figure 1, we found that SIRT4 played a role in PD models (MPTP-induced Parkinson’s mouse model and DJ-1 KO rat model) and subsequently revealed the possible mechanism of action of SIRT4 and potential molecular targets using quantitative proteomics and bioinformatics analyses in this study. Then our study further screened and validated that FABP4 in the PPAR signaling pathway is the most promising target of SIRT4 in the PD model based on the results from biochemical assays. Our results provide a therapeutic strategy targeting SIRT4 for the diagnosis and management of PD.

## 2. Materials and methods

### 2.1. Cell culture and cell transfection

The SH-SY5Y cell was purchased from Shanghai Zhong Qiao Xin Zhou Biotechnology Corp., Ltd. The cells were inoculated into DMEM/F12 (Sigma-HyClone, MO, USA, Cat#sh30023.01) containing 10% fetal bovine serum (Sigma-Gibco, MO, USA, Cat#16140089), 100 IU/ml penicillin, and 100 μg/ml streptomycin (Sigma-Gibco, MO, USA, Cat#15140148) after STR identification and mycoplasma detection and then incubated at 37^°^C with 5% CO_2_. Subculture was performed when the cells reached 80–90% confluence.

Human SIRT4 lentivirus to knock down (PLKD-CMV-EGFP-2A-PURO-U6-ShRNA) or overexpress (PRLENTI-EF1-EGFP-P2A-PURO-CMV-SIRT4-3xFLAG-WPRE) SIRT4 and mouse SIRT4-AAV virus to overexpress SIRT4 (pAAV-CMV-Sirt4-HA-EF1a-EGFP-tWPA) were purchased from Obio Technology (Shanghai) Corp., Ltd. Cells were plated in six-well plates at a density of 5 × 10^4^ cells per well and transfected with the SIRT4 lentivirus at an MOI of 80. The transfection rate was calculated by determining the fluorescence ratio after 72 h. Subsequently, SH-SY5Y cells were treated with 1500 μmol/L MPP^+^ (Sigma–Aldrich, MO, USA, Cat#D048) for 24 h, and then a PD cytotoxicity model was established. Cells were collected after 5 days to determine whether SIRT4 was overexpressed or knocked down. SH-SY5Y cells were divided into four groups: overexpression lentivirus control group (CN group), lentivirus-mediated SIRT4 overexpression group (G4 group), knockdown lentivirus control group (KX group), and lentivirus-mediated SIRT4 knockdown group (4X group).

### 2.2. Animals

Male C57BL/6 mice weighing 24–27 g and aged 10–12 weeks were chosen for the experiment, and were provided by the Fujian Medical University’s Experimental Animal Center. Two sgRNAs were designed to generate a 3.5 kb chromosomal deletion (exon 3∼5) at the Park7 locus in the rat genome. DJ-1 KO rats were purchased from Beijing Biositu Gene Biotechnology Co., Ltd. The animals used in the experiment were maintained in a standard breeding facility under pathogen-free conditions, with an ambient temperature of (22 ± 2)^°^C, relative humidity of 50–60%, a 12 h dark/light cycle, and access to water and food at all times. The experiment was approved by the Fujian Medical University Institutional Committee for Animal Care and Use with the ethics approval numbers FJMUIACUC 2021-0369 for mice and FJMUIACUC 2020-0016 for rats. All animal experiments were carried out in accordance with international animal biomedical research guidelines. The gene identification scheme for key familial Parkinson disease protein 7 knockout (DJ-1 KO) rats can be found in Supplementary Table 2.

### 2.3. Establishment of the MPTP-induced mouse model

**FIGURE 2:**
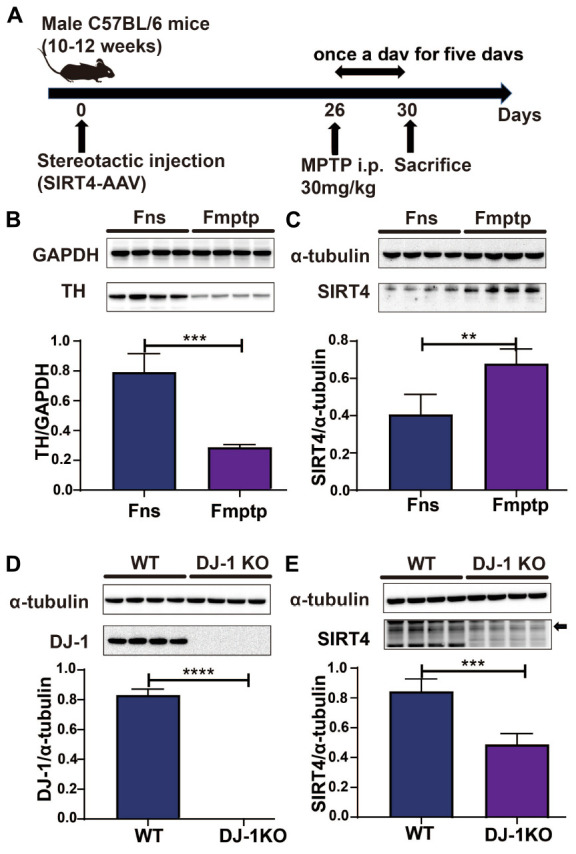
SIRT4 plays a role in a PD model. **(A)** Flow chart of stereotactic injection of SIRT4-AAV virus and the establishment of a mouse model of PD induced by MPTP. **(B)** Representative western blots and densitometric analysis for TH protein between groups. TH protein extracts from substantia nigra after mice were intraperitoneally injected with saline and MPTP for 5 days. GAPDH was used as a loading control (*n* = 4 mice per group). **(C)** Representative western blots and densitometric analysis for SIRT4 protein between groups. SIRT4 protein extracts from substantia nigra after mice were intraperitoneally injected with saline or MPTP for 5 days. Tubulin was used as a loading control (*n* = 4 mice per group). **(D,E)** Representative western blots and densitometric analysis for DJ-1 or SIRT4 protein between groups, respectively. DJ-1 or SIRT4 protein extracts from substantia nigra of WT rats or DJ-1 KO rats. Tubulin was used as a loading control (*n* = 4 mice per group). All results are depicted as means ± SEM. The comparison across groups was analyzed by *t*-test. **P < 0.01, ***P < 0.001, and ****P < 0.0001 compared with the control group. TH, tyrosine hydroxylase; SIRT4, NAD-dependent protein lipoamidase sirtuin-4, mitochondrial.

The mice used in the experiment were randomly assigned to receive either an intraperitoneal injection of normal saline (Fns group) or MPTP (Fmptp group). The mice were administered 30 mg/kg MPTP (Sigma–Aldrich, MO, USA, Cat#M0896) intraperitoneally once a day for five days to establish an MPTP-induced mouse model. The substantia nigra was removed from mice, and lysates were detected by western blotting and RT–qPCR on the first day after the last intraperitoneal injection. The specific operation process is depicted in Figure 2A.

### 2.4. Stereotactic injection

Male C57BL/6 mice aged 10–12 weeks underwent stereotactic injections. These animals were placed in a stereotactic frame and sedated with 2% isoflurane administered through a mask (oxygen flow rate of 2 L/min). Eye ointment was applied to the cornea and surrounding areas to ensure that they did not dry out during surgery. SIRT4-AAV and control-AAV were injected into the right striatum and right substantia nigra using a 2.5 L Hamilton syringe (32 G needle) in a stereotactic manner using the following coordinates of the right striatum and right substantia nigra: AP = +0.8 mm, ML = +2 mm and DV = −3.5 mm and AP = −2.6 mm, ML = +1.4 mm and DV = −4.5 mm from the bregma, respectively. Antibiotics and pain relievers were applied to the surface of the wound in each mouse. We waited for 10 min for virus infusion after the injection was done. One month after stereotactic injection of the virus, samples were collected for RT–qPCR and western blotting. Male C57BL/6 mice were divided into two groups: stereotactic injection of control AAV virus without intraperitoneal injection of MPTP group (KS group) and stereotactic injection of SIRT4-AAV virus without intraperitoneal injection of MPTP group (4S group). MPTP-induced Parkinson’s mouse model was divided into three groups: non-stereotactic injection group (Fmptp group), stereotactic injection of the control AAV group (kFmptp group), and stereotactic injection of the SIRT4-AAV group (4Fmptp group).

### 2.5. Western blot assay

After protein extraction, the protein concentration in the sample was determined by using the BCA method (Beyotime Biotechnology, Shanghai, China, Cat# P0012). Cell or tissue lysates were mixed with protein buffer and boiled for 10 min. Then, 30 μg from each group of samples was loaded in each lane and subjected to 10% sodium dodecyl sulfate–polyacrylamide gel electrophoresis (SDS-PAGE) (Beyotime Biotechnology, Shanghai, China, Cat# P0012A). Proteins with different molecular weights were separated at a voltage of 120 V and transferred to activated polyvinylidene difluoride (PVDF) membranes. After blocking with 5% skim milk powder for 2 h at room temperature, PVDF membranes were mixed with primary antibodies [SIRT4 (Proteintech, Wuhan, China, Cat#66543-1-IG), FABP4 (Proteintech, Wuhan, China, Cat#12802-1-AP), PPARγ (Proteintech, Wuhan, China, Cat#16643-1-AP), glyceraldehyde-3-phosphate dehydrogenase (GAPDH) (Abcam, Cambridge, UK, Cat#ab8245), α-tubulin (Abcam, Cambridge, UK, Cat# ab7291, DJ-1 (Abcam, Cambridge, UK, Cat#ab18257), β-actin (Abcam, Cambridge, UK, Cat#ab8227) and tyrosine hydroxylase (TH; Abcam, Cambridge, UK, Cat#ab112)]. After washing the membrane, it was soaked in ECL solution (solution A: solution B = 1:1) and reacted for 1–2 min. The membrane was then automatically exposed in a chemiluminescence instrument (PROTEIN SIMPLE, FluorChem M), and the gray value of the band was analysed using ImageJ software (*n* = 4).

### 2.6. RT-qPCR

RNA was extracted from each group of samples using TRIzol reagent (Invitrogen, Carlsbad, CA, Cat#15596018) for RT–qPCR analysis (*n* = 6). For reverse transcription, the EvoScript Universal Master one-step reverse transcription kit (Roche, Basel, Switzerland, Cat#7912439001) was used. Primers were designed based on the human/mouse gene sequence in NCBI GenBank, and their specificity was confirmed in advance using NCBI Blast. Supplementary Table 1 summarizes all primers used in this study. Fuzhou Shangya Biotechnology Co., Ltd. synthesized the primers and tested their quality. FastStart Universal SYBR Green Master Mix (ROX) (Roche, Basel, Switzerland, Cat# 04913914001) and a 7,500 PCR instrument (Applied Biosystems) were used for relative quantification with the following thermal cycling program: 55^°^C for 2 min; 95^°^C for 10 min; 40 cycles of 95^°^C for 15 s and 60^°^C for 60 s; 95^°^C for 15 s; 60^°^C for 60 s, and 95^°^C for 15 s. The expression of the target mRNAs in all the samples was normalized to that of β-actin using the 2^–ΔΔ*Ct*^ method.

### 2.7. Quantitative proteomics: Sample preparation, protein digestion, TMT labeling, and LC–MS/MS analysis

SH-SY5Y cells transfected with empty virus (CN group) and SIRT4 overexpression lentivirus (G4 group) were used in this study. An equal number of samples from each group were chosen for enzymolysis and the appropriate amount of standard protein was added. Dithiothreitol (DTT) was added to the samples to a final concentration of 5 mM, and then the concentration was reduced by incubating the samples at 56^°^C for 30 min. Subsequently, iodoacetamide (IAA) was added to a final concentration of 11 mM, and the mixture was incubated at room temperature in the dark for 15 min. In addition, triethylamine-carbonic acid buffer solution (TEAB) was added to dilute the urea to a concentration lower 2 M. Finally, trypsin was added at a 1:50 (protease:protein, m/m) ratio, and enzymolysis was performed overnight. Trypsin was then added at a ratio of 1:100 (protease:protein, m/m), and the enzymolysis reaction was continued for 4 h.

The trypsin-digested peptides were desalted with Strata X (Phenomenex) and lyophilized under a vacuum. Peptides were dissolved in 0.5 M TEAB and labeled according to the instructions provided with the labeling kit. The peptides were separated using an ultrahigh-performance liquid system, injected into nanospray ionization source, and analysed by mass spectrometry. The ion source voltage was set to 2.0 kV, and the parent ion of the peptide as well as its secondary fragments were detected and analysed with a high-resolution Orbitrap system.

### 2.8. Database analysis

WoLF PSORT software was used in this study to predict the protein structure and subcellular localization. For protein domain annotations, InterProScan, a software package that searches the InterPro database based on sequence alignment was used. Protein GO annotation data were primarily derived from the UniProt-GOA database. The ID of the protein was first converted into the ID of the UniProtKB database, and then the relevant GO annotation information was retrieved from the UniProt-GOA based on the UniProtKB ID. If some identified proteins were not annotated in the database, their GO classification was annotated using the InterProScan homology alignment method. Protein metabolic pathways were annotated in the Kyoto Encyclopedia of Genes and Genomes (KEGG) database. KAAS, a KEGG online service tool, was used to annotate the proteins and obtain the KO number of the proteins corresponding to the KEGG database. The KO number was then mapped to a specific biological route using KEGG Mapper, a KEGG online service tool. Fisher’s exact test was utilized for the enrichment analysis of differentially expressed proteins identified from the different comparisons based on GO terms, KEGG pathways, and protein domains, and the corresponding *P*-values were calculated. The related functions of differentially expressed proteins identified from the different comparisons were clustered by hierarchical clustering analysis. The protein–protein interactions (PPIs) of differentially expressed proteins were extracted based on a confidence score greater than 0.7 after comparing the database numbers or protein sequences of the differentially expressed proteins selected from the sets identified from the different comparisons with the STRING (v.10) database. The interactive network of differentially expressed genes and proteins was then visualized using the R package “networkD3” tool. The gene set enrichment analysis (GSEA) algorithm was created by the Broad Institute. The enrichment results for differentially expressed proteins were filtered based on a *P*-value < 0.05.

### 2.9. Statistical analysis

The parameters used for the GO enrichment analysis were as follows: background, the number of identified proteins in the GO classification; mapping, the number of differentially expressed proteins in the GO classification; all Mapping, the number of all differentially expressed proteins annotated by GO; all Background: the number of all identified proteins annotated by GO; fold enrichment, the ratio of the number of expressed proteins in the specific GO classification to the number of identified proteins in the classification. If mapping = a, background = b, all mapping = c, and all background = d, fold enrichment = (a/c)/(b/d). The KEGG pathway and protein domain enrichment analyses were performed using similar methods. GraphPad Prism 7.0 software (San Diego, CA, USA) was used for the statistical analyses. Student’s *t*-test was used to analyse the differences between two groups, ANOVA was used to analyse the differences among multiple groups, and *P* < 0.05 was considered to indicate a significant difference.

## 3. Results

### 3.1. SIRT4 plays a role in a PD model

**FIGURE 3:**
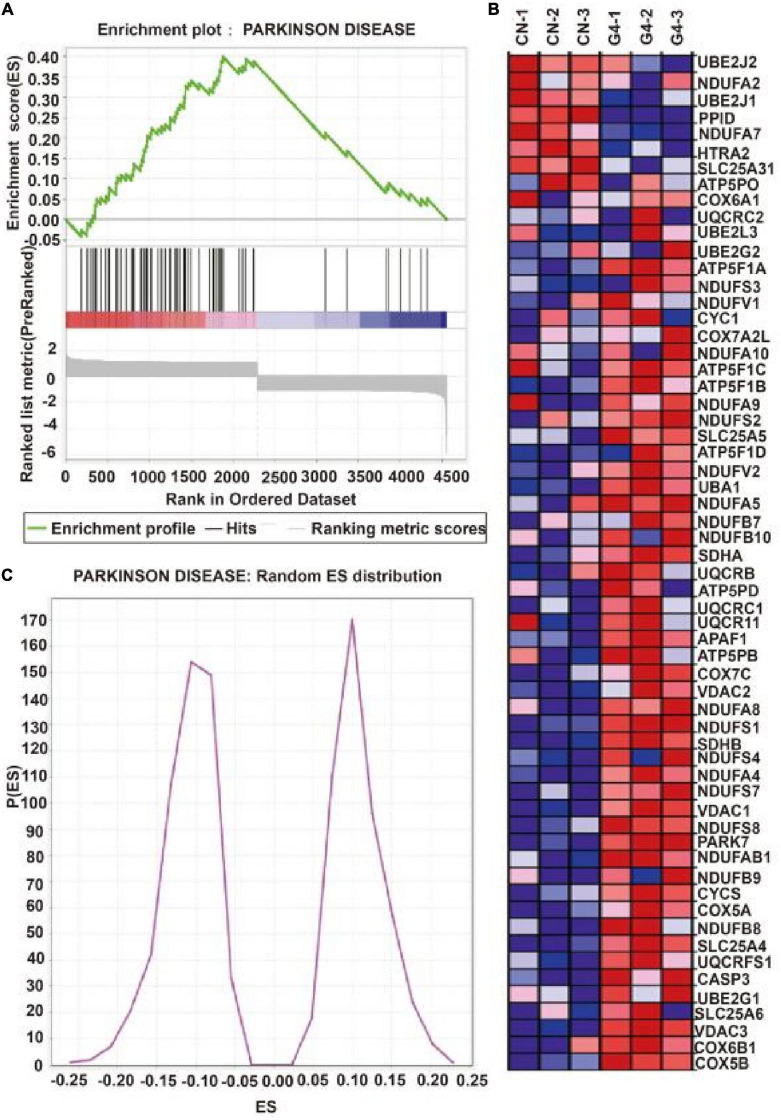
Gene set enrichment analysis of differentially expressed proteins after SIRT4 overexpression in SH-SY5Y cells. **(A)** Gene set enrichment analysis of differentially expressed protein after overexpression of SIRT4 in Parkinson’s disease pathway. The graph is divided into three parts. The first part is the line graph of Enrichment Score (ES). The horizontal axis is all proteins (arranged from high to low according to the protein expression). The vertical axis is the running ES corresponding to the proteins under the functional set. The second part is hit information. Whether the proteins in the sorted list exist in the functional set is marked by lines. The lower color bar indicates the corresponding expression of proteins. The third part is the distribution map of ratios of all proteins. **(B)** Random ES distribution in Parkinson’s disease pathway. **(C)** The heat map of differentially expressed proteins in Parkinson’s disease pathway after gene set enrichment analysis.

Figure 2A depicts the establishment of the MPTP-induced mouse model. TH protein expression was significantly lower in the MPTP-induced mouse model than in the control group (*P* < 0.05), indicating that the MPTP-induced mouse model was successfully established (Figure 2B). In this model, decreases in TH protein expression were accompanied by increases in the expression of SIRT4 protein, and the correlation was significant (Figure 2C). Compared with the WT group, DJ-1 was completely knocked out in the DJ-1KO rat model (Figure 2D) and SIRT4 protein expression was decreased (*P* < 0.05) (Figure 2E). PD is caused by the interaction of genes and the environment. These results imply that both the PD model induced by environmental toxins and the early-onset PD model induced by DJ-1 gene deletion are accompanied by obvious changes in SIRT4 protein expression. Furthermore, a GSEA of differentially expressed proteins in SH-SY5Y cells transfected with the empty virus (CN group) compared with cells transfected with SIRT4 overexpression lentivirus (G4 group) revealed the number of functional sets enriched by upregulated/downregulated proteins and the number of functional sets enriched at different significance thresholds. A positive enrichment score (ES) indicates that the functional set is enriched at the top of the list, namely, an upregulated protein is enriched in the functional set, and a negative value indicates that a downregulated protein is enriched in the functional set. NES is the abbreviation for the normalized enrichment score. The main results for the NES values (G4/CN) are summarized in Supplementary Table 3. In the table, the NES value of the PD pathway was 3.666, indicating that this pathway ranked fourth among all the pathways (Supplementary Table 3). Figure 3 also shows the protein’s position in the ordered list, the corresponding expression level, and the ratio from the comparison. Furthermore, Figure 3C displays a heatmap of the related proteins enriched in the PD pathway. SIRT4 overexpression increases the expression of related proteins in the PD pathway, which play a protective role in PD. In summary, a GSEA of the proteomics results from the *in vitro* model supports the notion that SIRT4 significantly participates in PD by exerting protective effects.

### 3.2. Identification of differentially expressed proteins between the SIRT4 group and control group in SH-SY5Y cells

**FIGURE 4:**
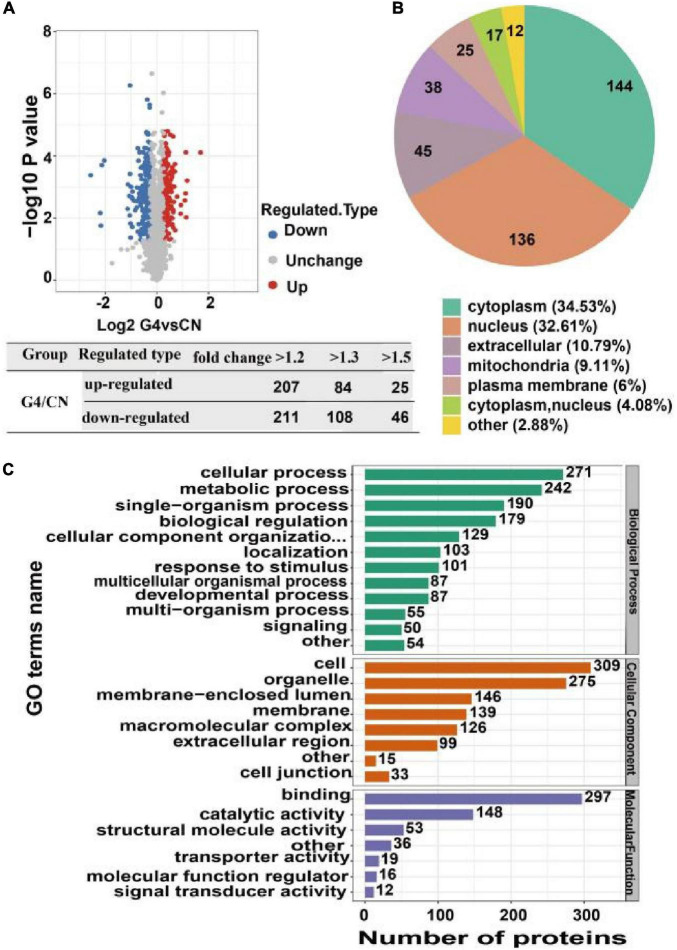
Identification, subcellular distribution and Gene Ontology annotation of differentially expressed proteins in SH-SY5Y cells after SIRT4 overexpression. **(A)** Volcanic map of differentially expressed proteins identified from the comparisons. Each dot in the figure represents a protein. The red dots represent upregulated proteins, the blue dots represent downregulated proteins, and the gray dots represent upregulated proteins with no significant difference. The horizontal axis shows the protein expression ratio after log2 conversion. The vertical axis shows the *P*-value of the difference determined by the statistical analysis after conversion by –log10. The number of differentially expressed proteins based on different fold changes is also summarized. **(B)** Pie chart of the subcellular distribution of differentially expressed proteins. Different colors indicate different subcellular locations. The legend indicates the name and proportion of the subcellular localization corresponding to the color. **(C)** Bar graph of Gene Ontology annotation. The vertical axis represents the secondary classification of GO terms. The horizontal axis represents the number of differentially expressed proteins. The color of the bar graph represents three different first-level GO classifications: biological process (green), molecular function (purple), and cellular component (orange).

The quantitative proteomics results revealed that the G4 group had 207 upregulated proteins and 211 downregulated proteins compared with the CN group based on a fold change in protein expression higher than 1.2. The G4 group had 84 upregulated proteins and 108 downregulated proteins compared with the CN group based on a fold change in protein expression greater than 1.3. In addition, the G4 group had 25 upregulated proteins and 46 downregulated proteins compared with the CN group with fold change in protein expression higher than 1.5 (Figure 4A). Furthermore, our findings revealed that 30 upregulated and 30 downregulated proteins exhibited the most notable changes (Supplementary Figures 4, 5).

### 3.3. Functional annotation of differentially expressed proteins between the SIRT4 group and control group in SH-SY5Y cells

Gene Ontology (GO) annotation, biological pathways, protein domains, subcellular structure localization, and Clusters of Orthologous Groups/EuKaryotic Orthologous Groups (COG/KOG) categories are all aspects of protein functional annotation. GO annotation is a major bioinformatics analysis method and is classified into three categories: biological processes (BPs), cellular components (CCs), and molecular functions (MFs). Compared with the levels in the CN group, the proteins whose expression changed after SIRT4 overexpression were mainly involved in the following BPs: cellular processes, biological regulation and single biological processes, metabolic processes, and responses to stimulation. In terms of CCs, the majority of the differentially expressed proteins were located in cells (27%), organelles (24%), and membrane closed cavities (12.78%). In terms of MFs, differentially expressed proteins were mainly enriched in binding (50%) and catalytic activity (25.38%) (Figure 4C).

The analysis of the subcellular localization of the differentially expressed proteins revealed that they were mainly located in the cytoplasm (34.53%), nucleus (32.61%), and extracellular space (10.79%) (Figure 4B).

**FIGURE 5:**
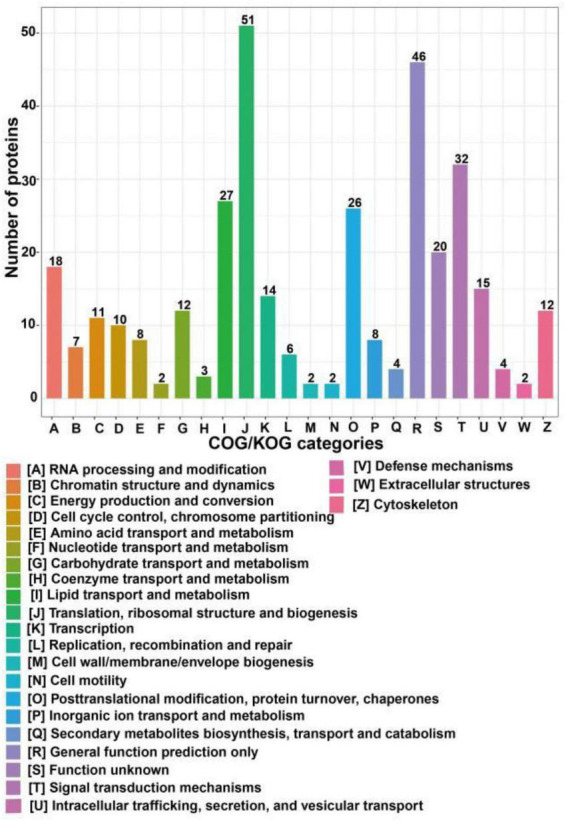
COG/KOG categories of differentially expressed proteins after SIRT4 overexpression in SH-SY5Y cells. The vertical axis of the bar graph is the COG classification, and the horizontal axis is the number of differential proteins.

COG/KOG categories indicated that the differentially expressed proteins following SIRT4 overexpression primarily affected biological processes such as ribosome structure and biogenesis, lipid transport and metabolism, signal transduction mechanism, translation process and posttranslational modification, protein turnover, molecular chaperone, RNA processing, modification, chromosome allocation, cellular endocrine and vesicle transport, and carbohydrates involved in amino acid transport and metabolism (Figure 5).

### 3.4. Functional enrichment analysis of differentially expressed proteins between the SIRT4 group and control group in SH-SY5Y cells

**FIGURE 6:**
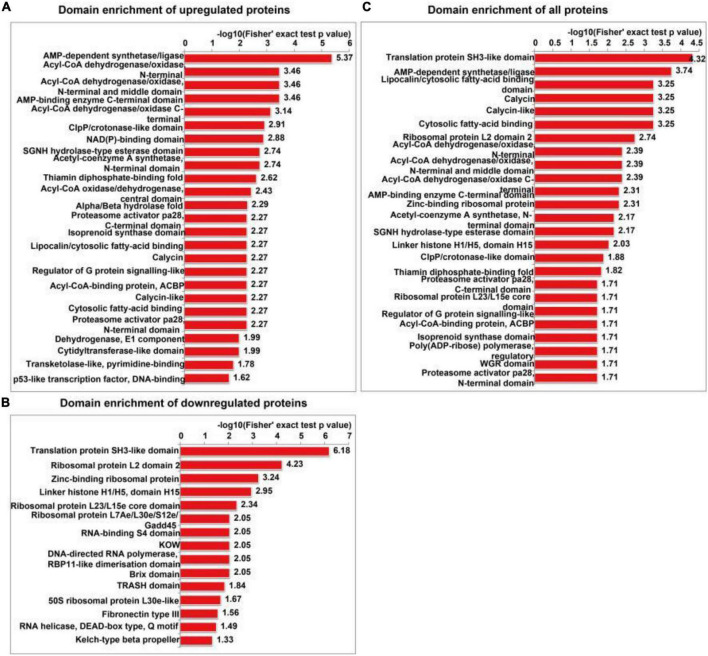
Protein domain enrichment analysis of differentially expressed proteins in SH-SY5Y cells after SIRT4 overexpression. **(A–C)** Represents the visual results from the protein domain enrichment analysis of upregulated proteins, downregulated proteins and differentially expressed proteins (including upregulated and downregulated proteins). The vertical axis shows the GO classification, and the horizontal axis shows Fisher’s exact *P*-value after logarithmic conversion. The longer the bar graph is, the more significantly the differentially expressed proteins are enriched in the corresponding classification or function.

The functional enrichment analyses of differentially expressed proteins included GO, KEGG pathway and protein domain enrichment analyses. AMP-dependent synthetase/ligase, acyl-CoA dehydrogenase/oxidase, N-terminal, acyl-CoA dehydrogenase/oxidase, and intermediate domains were the most commonly upregulated protein domains (Figure 6A). The translation protein SH3-like domain, ribosomal protein L2 domain 2, and zinc-binding ribosomal protein were the most commonly downregulated protein domains (Figure 6B). All the enriched protein domains mainly included the translation protein SH3-like domain, AMP-dependent synthetase/ligase and lipocalin/cytoplasmic fatty acid binding domain (Figure 6C). In summary, SIRT4 primarily affects the translation protein SH3-like domain.

**FIGURE 7:**
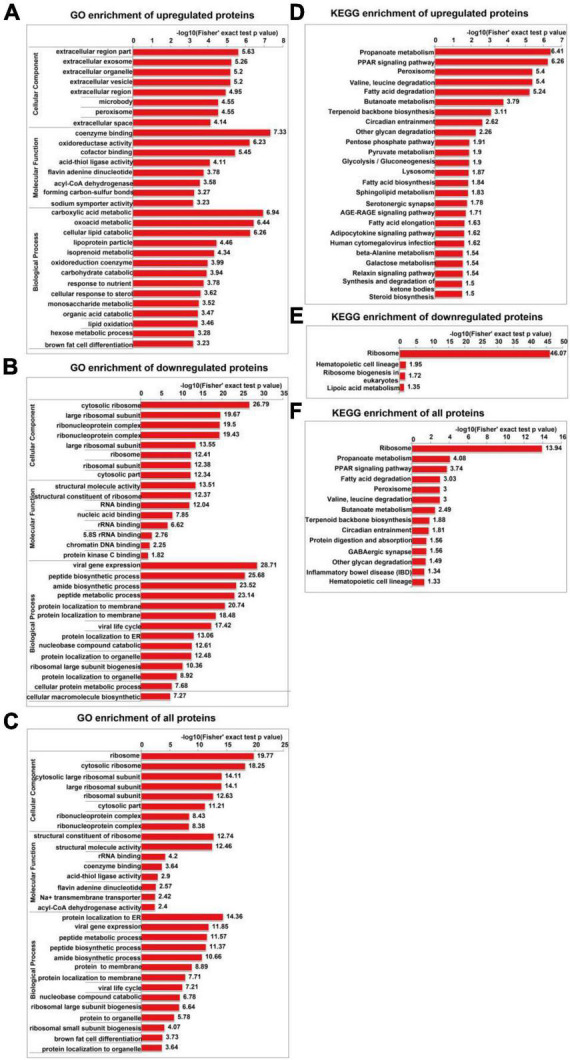
GO and KEGG enrichment analyses of differentially expressed proteins in SH-SY5Y cells after SIRT4 overexpression. The data used for the GO and KEGG enrichment analyses were derived from all the identified proteins. **(A–C)** Represents the visual results from the GO enrichment analysis of upregulated proteins, downregulated proteins and differentially expressed proteins (including both upregulated and downregulated proteins). **(D–F)** Show the visual results from the KEGG enrichment analysis of upregulated proteins, downregulated proteins and differentially expressed proteins (including both upregulated and downregulated proteins). The vertical axis is the GO classification, and the horizontal axis is the Fisher’s exact *P*-value after logarithmic conversion. The longer the bar graph is, the more significantly the differentially expressed proteins are enriched in the corresponding classification or function.

**FIGURE 8:**
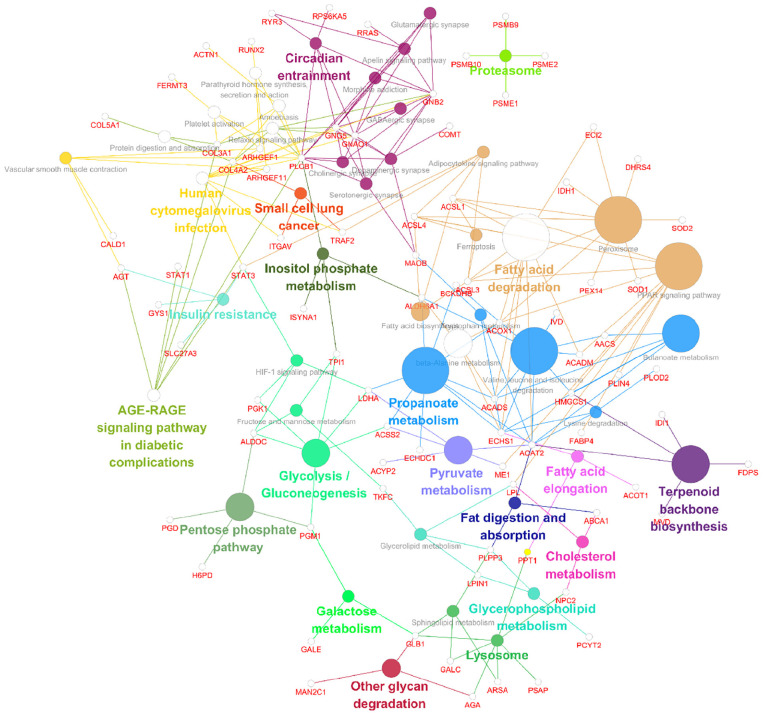
Data visualizations of the results from the KEGG enrichment analysis of differentially expressed genes and proteins in SH-SY5Y cells after SIRT4 overexpression with ClueGO. A KEGG enrichment analysis of differentially expressed proteins in SH-SY5Y cells after SIRT4 overexpression was performed using ClueGO. The figure clearly shows the main proteins in the enriched pathways and the connection between A protein and different pathways. The size of the circle represents the number of different proteins.

The GO enrichment analysis of upregulated proteins included carboxylic acid metabolism, oxyacid metabolism, and cellular lipid catabolism (Figure 7A). The GO enrichment analysis of downregulated proteins included viral gene expression, peptide biosynthesis, and amide biosynthesis (Figure 7B). The GO enrichment analysis of all differentially expressed proteins included protein localization to the endoplasmic reticulum, viral gene expression, and peptide metabolism (Figure 7C). In summary, the GO terms enriched in the altered proteins were primarily associated with protein localization to the endoplasmic reticulum and viral gene expression. The KEGG enrichment analysis of upregulated proteins mainly included propionic acid metabolism, the PPAR signalling pathway, peroxisome, valine, leucine, and isoleucine degradation, and fatty acid degradation (Figure 7D). The KEGG enrichment analysis of downregulated proteins mainly included the ribosome, haematopoietic lineage, ribosome biogenesis in eukaryotes, and lipoic acid metabolism (Figure 7E). The KEGG enrichment analysis of all differentially expressed proteins included ribosome, propionic acid metabolism, PPAR signaling pathway, fatty acid degradation and peroxisome, valine, leucine, and isoleucine degradation (Figure 7F). Figure 8 depicts the main enriched KEGG pathways and the corresponding altered proteins.

### 3.5. Protein-protein interaction (PPI) network analysis of differentially expressed proteins between the SIRT4 group and control group in SH-SY5Y cells

**FIGURE 9:**
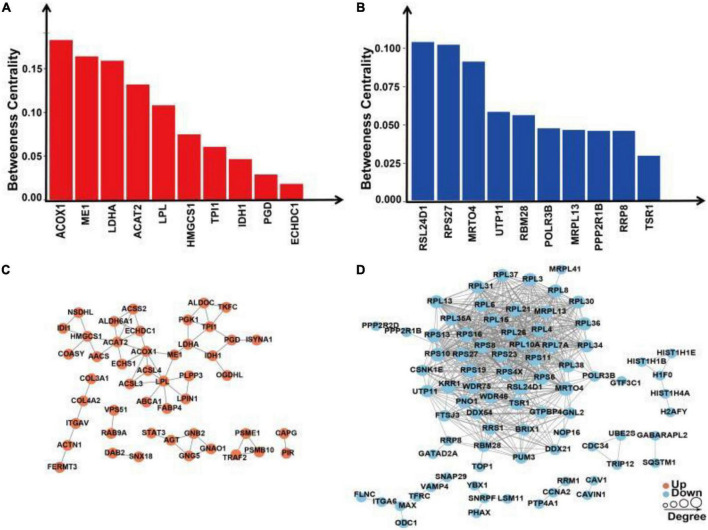
Protein–protein interaction network analysis of differentially expressed proteins in SH-SY5Y cells after SIRT4 overexpression. **(A)** Histogram of the top 10 active proteins in the PPI network analysis of upregulated proteins according to the betweenness centrality. **(B)** Histogram of the top 10 active proteins in the PPI network analysis of downregulated proteins according to the betweenness centrality. **(C)** Visual display of the PPI network analysis of upregulated proteins. **(D)** Visual display of the PPI network analysis of downregulated proteins. A network of interactions from the STRING database with a confidence score >0.7 (high confidence) was visualized in Cytoscape. The betweenness centrality was calculated based on the Cytoscape plug-in CytoNCA. The size of the circle represents the number of proteins interacting with the corresponding differentially expressed proteins.

We extracted the interactions of different proteins with a confidence score >0.7 after comparing all differentially up/downregulated proteins with the STRING (v.10) database. Betweenness centrality is a parameter reflecting the activity and importance of proteins in the protein–protein interaction network. The ACOX1, ME1, LDHA, ACAT2, LPL, HMGCS1, TPI1, IDH1, PGD, and ECHDC1 proteins were identified in the PPI analysis for upregulated proteins sorted by the ratio of betweenness centrality (Figure 9A). The RSL24D1, RSP27, MRT04, UTP11, RBM28, POLR3B, MRPL13, PPP2R1B, RRP8, and TSR1 proteins were identified in the PPI analysis for downregulated proteins sorted by the ratio of betweenness centrality (Figure 9B).

**FIGURE 10:**
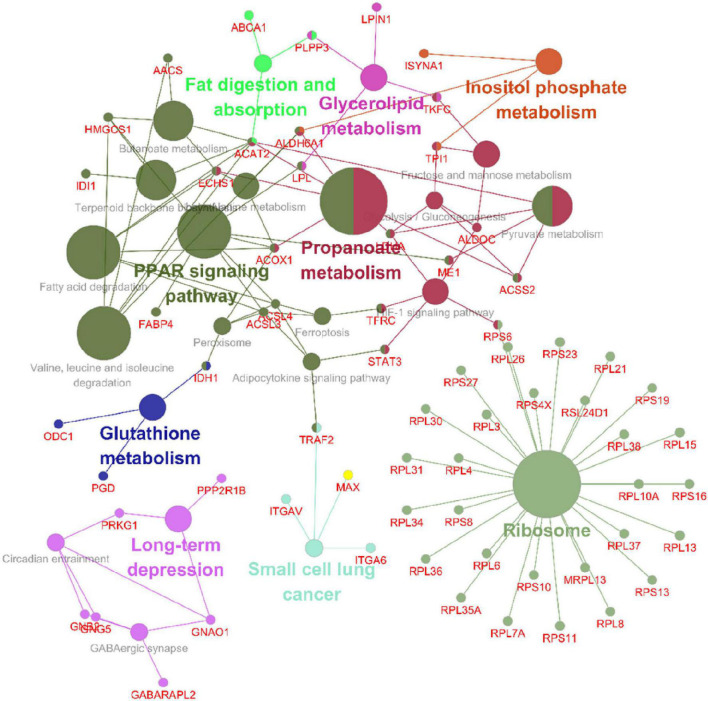
KEGG enrichment analysis of all interacting proteins after SIRT4 overexpression in SH-SY5Y cells with ClueGO. The figure clearly shows the main proteins in the enrichment pathway and the connection between the same protein and different pathways. The size of the circle represents the number of different proteins.

Following the change in SIRT4 expression, the activity of these molecules changes and are regarded as promising potential targets for SIRT4 to play a role in the PD model, which merits further investigation in follow-up studies. Furthermore, we conducted a KEGG enrichment analysis of these active proteins and discovered that they were primarily enriched in the ribosome, the PPAR signaling pathway, propionic acid metabolism, inositol phosphate metabolism, lipid metabolism, and glutathione metabolism. Figure 10 depicts the main enriched KEGG pathways of these active proteins and the corresponding altered proteins.

### 3.6. Among the 25 candidate targets, only the transcription of FABP4 can be regulated by SIRT4 in SH-SY5Y cells or SH-SY5Y cells with MPP^+^ intervention

Regardless of whether the results were obtained from the enrichment analysis of differentially expressed proteins or related proteins following the PPI network analysis, the mechanism of action of SIRT4 is primarily anchored in the ribosome pathway, the PPAR signaling pathway, the propionic acid metabolism pathway and the peroxisome pathway. RPL6 is the protein in the ribosome pathway exhibiting the most significant change. FABP4 is the protein in the PPAR pathway exhibiting the most significant change. BCKDHB and ACSS2 are the most significantly altered proteins in the propionic acid metabolism pathway. SOD1 is the protein in the peroxisome pathway displaying the most significant change. We identified the above proteins, the top 10 active proteins in the PPI network analysis for upregulated proteins as well as the top 10 active proteins in the PPI network analysis for downregulated proteins as potential targets for SIRT4 regulation.

**FIGURE 11:**
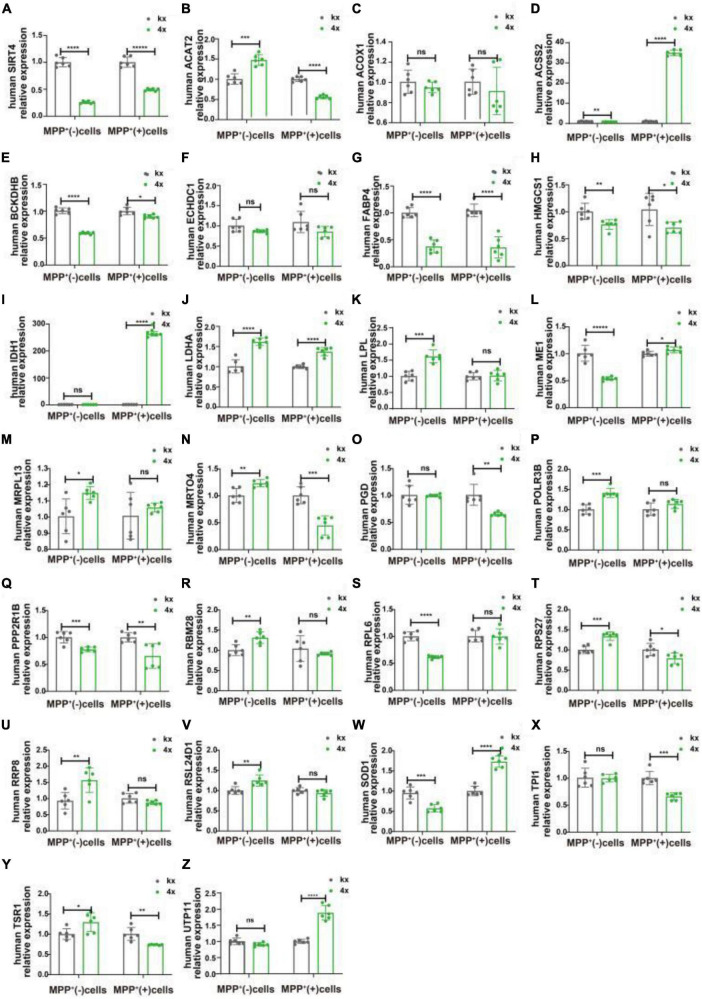
The mRNA expression of 25 potential targets in SH-SY5Y cells or MPP + -treated SH-SY5Y cells after knocking down SIRT4. **(A)** The mRNA expression of SIRT4 in SH-SY5Y cells or MPP^+^-treated SH-SY5Y cells after knocking down SIRT4. **(B–Z)** Compared with the control group, the mRNA expression of 25 potential targets in SH-SY5Y cells or MPP^+^-treated SH-SY5Y cells after knocking down SIRT4, respectively. The different mRNA expressions were normalized by β-ACTIN (*n* = 6 per group). Results are depicted as means ± SEM. *P < 0.05, **P < 0.01, ***P < 0.001, and ****P < 0.0001 compared with the control group.

**FIGURE 12:**
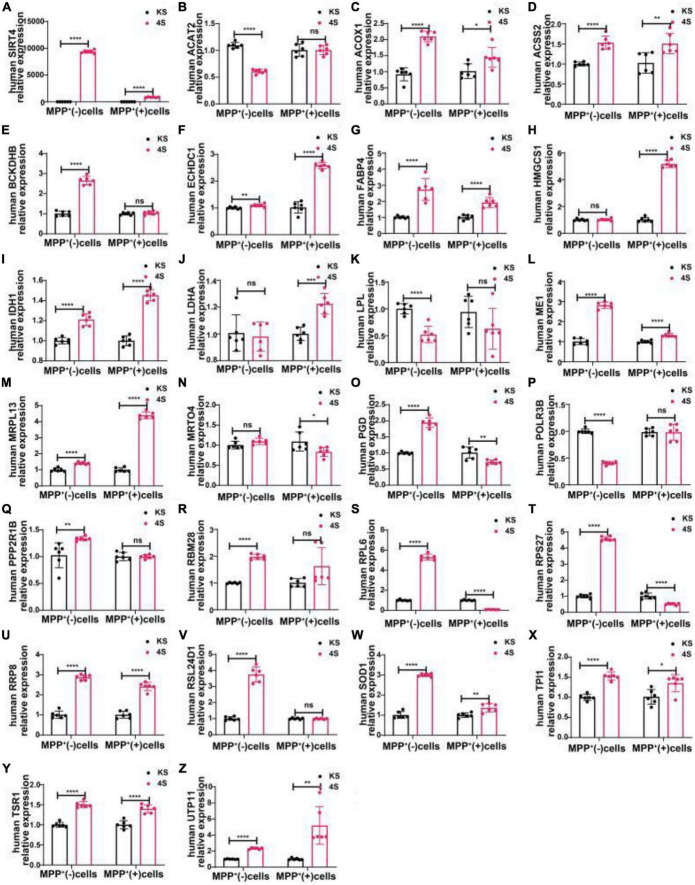
The mRNA expression of 25 potential targets in SH-SY5Y cells or MPP^+^-treated SH-SY5Y cells after SIRT4 overexpression. **(A)** The mRNA expression of SIRT4 in SH-SY5Y cells or MPP^+^-treated SH-SY5Y cells after SIRT4 overexpression. **(B–Z)** Compared with the control group, the mRNA expression of 25 potential targets in SH-SY5Y cells or MPP^+^-treated SH-SY5Y cells after SIRT4 overexpression, respectively. The different mRNA expressions were normalized by β-ACTIN (*n* = 6 per group). Results are depicted as means ± SEM. *P < 0.05, **P < 0.01, ***P < 0.001, and ****P < 0.0001 compared with the control group.

We transfected SH-SY5Y cells or SH-SY5Y cells with MPP^+^ intervention by lentiviruses that overexpressed or knocked down SIRT4 to observe the expression of these proteins and further assessed the most promising regulatory targets (Figures 11, 12). Table 1 summarizes the related results and clearly shows that only FABP4 transcription increased after SIRT4 overexpression and decreased after the knockdown of SIRT4 expression, indicating that it is regulated by SIRT4 in both SH-SY5Y cells or SH-SY5Y cells intervented with MPP^+^.

**TABLE 1:** The changes of 25 potential targets in mRNA expression under different intervention conditions in SH-SY5Y cells and wild-type (C57) mice.

		Compared with the corresponding group				
Gene	Gene description	SIRT4 overexpression in SH-SY5Y	Knock-down of SIRT4 in SH-SY5Y	SIRT4 overexpression in MPP+ intervention SH-SY5Y cell	Knock-down of SIRT4 in MPP+ intervention SH-SY5Y cell	SIRT4 overexpression in normal mice
		G4 group	KX group	G4+M group	KX+M group	4S
SIRT4	NAD-dependent protein lipoamidase sirtuin-4	↑	↓	↑	↓	↑
RPL6	60S ribosomal protein L6	↑	↓	↓	ns	ns
LPL	Lipoprotein lipase	↓	↑	↓	ns	↓
PGD	6-phosphogluconate dehydrogenase, decarboxylating	↑	**ns**	↓	↓	↑
HMGCS1	Hydroxymethylglutaryl-CoA synthase, cytoplasmic	**ns**	↓	↑	↓	↑
TPI1	Triosephosphate isomerase	↑	ns	↑	↓	ns
BCKDHB	2-oxoisovalerate dehydrogenase subunit beta	↑	↓	**ns**	↓	↓
ECHDC1	Ethylmalonyl-CoA decarboxylase	**ns**	ns	↑	ns	↓
UTP11	Probable U3 small nucleolar RNA-associated protein 11	↑	**ns**	↑	↑	↑
POLR3B	DNA-directed RNA polymerase III subunit RPC2	↓	↑	**ns**	ns	ns
ACSS2	Acetyl-coenzyme A synthetase, cytoplasmic	↑	↓	↑	↑	↑
RSL24D1	Probable ribosome biogenesis protein RLP24	↑	↑	**ns**	ns	ns
RBM28	RNA-binding protein 28	↑	↑	**ns**	ns	↓
MRPL13	39S ribosomal protein L13, mitochondrial	↑	↑	↑	ns	ns
MRT04	mRNA turnover protein 4 homolog	**ns**	↑	↓	↓	↑
PPP2R1B	Serine/threonine-protein phosphatase 2A 65 kDa regulatory subunit A beta isoform	↑	↓	ns	↓	↓
AC0X1	Peroxisomal acyl-coenzyme A oxidase 1	↑	**ns**	↑	ns	↓
S0D1	Superoxide dismutase [Cu-Zn]	↑	↓	↑	↑	↓
ME1	NADP-dependent malic enzyme	↑	↓	↑	↑	ns
RPS27	40S ribosomal protein S27	↑	↑	↓	↓	↓
ACAT2	Acetyl-CoA acetyltransferase, cytosolic	↓	↑	**ns**	↓	ns
LDHA	L-Lactate dehydrogenase A chain	**ns**	↑	↑	↑	ns
FABP4	Fatty acid-binding protein, adipocyte	↑	↓	↑	↓	↓
IDH1	Isocitrate dehydrogenase [NADP] cytoplasmic	↑	**ns**	↑	↑	↓
RRP8	Ribosomal RNA-processing protein 8	↑	↑	↑	ns	ns
TSR1	Pre-rRNA-processinq protein TSR1 homolog	↑	↑	↑	↓	↓

Red font represents among the 25 molecules, FABP4 as the most potential target.

### 3.7. FABP4 in the PPAR signaling pathway is a potential target in the MPTP-induced PD mouse model

**FIGURE 13:**
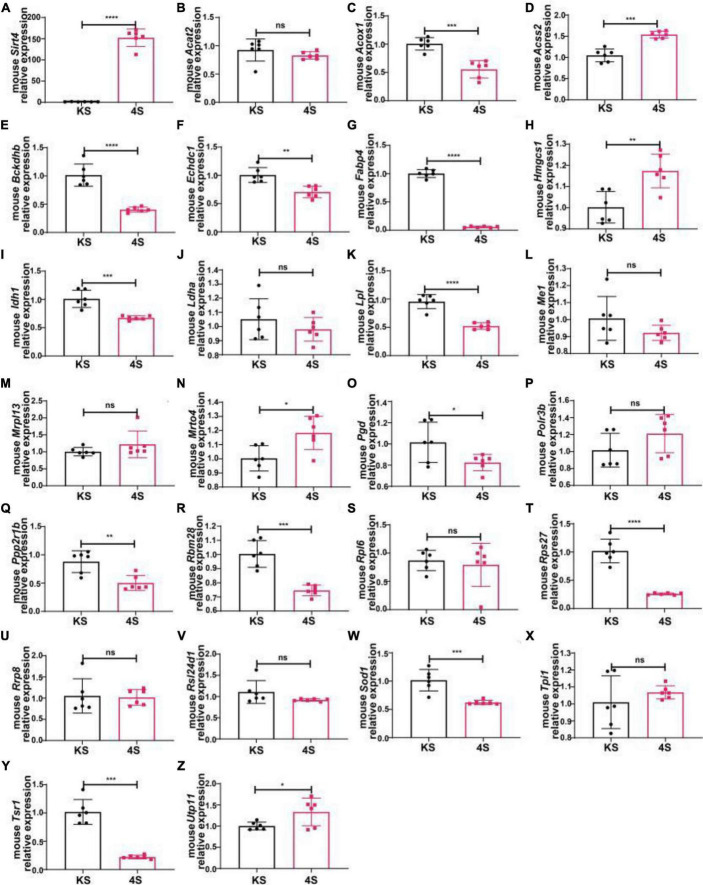
The mRNA expression of 25 potential targets in wild-type (C57) mice after SIRT4 overexpression. **(A)** The mRNA expression of SIRT4 in the substantia nigra of wild-type (C57) mice after stereotactic injection of SIRT4-AAV. **(B–Z)** The mRNA expression of 25 potential targets in the substantia nigra of wild-type (C57) mice after stereotactic injection of SIRT4-AAV, respectively. The different mRNA expression levels were normalized to that of β-actin (*n* = 6 per group). The results are depicted as the means ± SEMs. *P < 0.05, **P < 0.01, ***P < 0.001, and ****P < 0.0001 compared with the control group.

**FIGURE 14:**
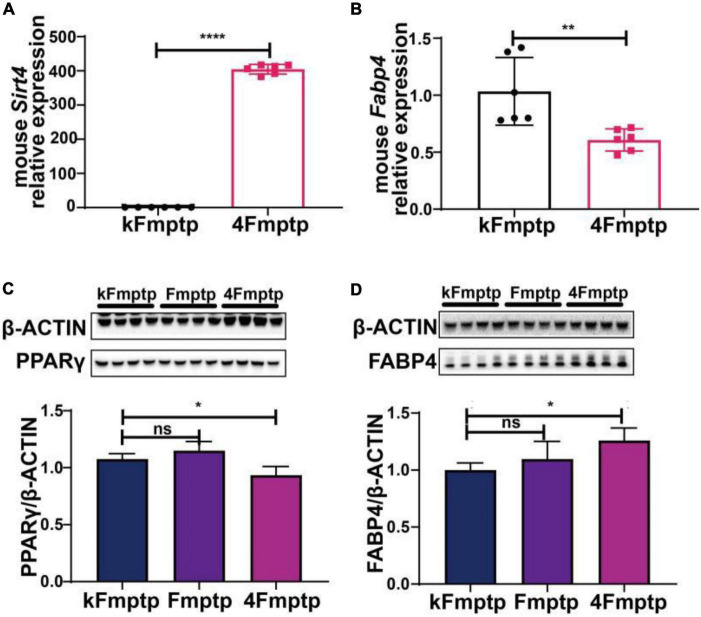
SIRT4 overexpression in the substantia nigra of MPTP-induced Parkinson’s mouse model can induce the changes of FABP4 and PPARγ. **(A)** The mRNA expression of SIRT4 in the substantia nigra of MPTP-induced Parkinson’s mouse model after stereotactic injection of SIRT4-AAV. **(B)** The mRNA expression of FABP4 in the substantia nigra of MPTP-induced Parkinson’s mouse model after stereotactic injection of SIRT4-AAV. **(C,D)** Representative western blots and densitometric analysis for FABP4 γ PPAR protein between groups, respectively. FABP4 γ PPAR protein extracts from the substantia nigra of MPTP-induced Parkinson’s mouse model after stereotactic injection of SIRT4-AAV or the control group. The different protein expressions were normalized by β-Actin (*n* = 6 per group). Results are depicted as means ± SEM. *P < 0.05, **P < 0.01, and ****P < 0.0001 compared with the control group. FABP4, Fatty acid binding protein 4; PPARγ, Peroxisome proliferator-activated receptor gamma.

We selected male C57BL/6 mice for the stereotactic injection of SIRT4-AAV to observe the changes in the mRNA expression of 25 potential molecules in mice (4S group), and the results are shown in Figure 13 and Table 1. Compared with the control empty virus group (KS group), we discovered that the mRNA level of FABP4 decreased after SIRT4 overexpression, and the difference was significant (*P* < 0.05). We administered stereotactic injection of SIRT4-AAV into the substantia nigra and striatum of the MPTP mouse model (4Fmptp group). After SIRT4 overexpression, FABP4 expression decreased significantly at the mRNA level (*P* < 0.05) compared with that in the control empty virus group (kFmptp group). At the protein level, the expression of FABP4 and PPARγ increased compared with that in the control empty virus group (kFmptp group) (*P* < 0.05) (Figure 14). Our results support the hypothesis that FABP4 in the PPAR signalling pathway is a potential target in the mouse model of MPTP-induced PD.

## 4. Discussion

NAD^+^ is a key metabolite involved in cellular bioenergetics, genomic stability, mitochondrial homeostasis, adaptive stress responses, and cell survival (Montaigne et al., 2021). The SIRT family is a group of NAD^+^-dependent deacetylases that have become a focus of interest in recent years due to their multiple regulatory functions, especially in aging and metabolism. Sirtuins act in different cellular substructures: they deacetylate histones and several transcriptional regulators in the nucleus as well as specific proteins in the cytoplasm, mitochondria, and other organelles, thus acting as key regulators of energy homeostatic networks (Houtkooper et al., 2012). Interactions have also been observed between different SIRTs, and thus a comprehensive understanding of the function of the SIRT family in several pathways is thus crucial (Singh et al., 2018). The function of mitochondria in the pathogenesis/aetiology of PD is specifically prominent, which includes damage to the mitochondrial respiratory chain complex as an important feature of PD, the extensive involvement of the mitochondrial genome in its progression, the interactions between α-syn and mitochondrial function, the disruption of mitochondrial homeostasis by Parkinson’s disease-associated pathogenic genes, and the drugs targeting mitochondrial mechanisms to improve its prognosis (Grünewald et al., 2019). Mitochondrial SIRTs are anchored in the mitochondria and show the advantages of influencing neurodegenerative diseases through posttranslational modifications of substrate proteins to regulate the activity and biological functions of mitochondrial proteins, making them of great interest to us (He et al., 2022). SIRT4 is the least studied SIRT, thus necessitating its exploration as a new potential target and the determination of its role in PD. This study focused on whether SIRT4 was involved in PD models, and we innovatively investigated possible pathways and molecular targets of SIRT4 by performing quantitative proteomics with TMT Labeling. PD is considered to result from genes, environment, and gene-environment interactions. The MPTP mouse model is a typical drug toxicity model of PD, and the DJ-1KO rat is a PD model with deletion of the DJ-1 gene associated with oxidative stress (Díaz-Casado et al., 2016). In our study, SIRT4 levels were altered in both the MPTP-induced PD mouse model and the DJ-1KO rat model, reflecting that SIRT4 may be involved in the progression of PD. In addition, we selected SH-SY5Y cell as the cell model, which exhibits various properties of dopaminergic neurons, such as tyrosine hydroxylase, dopamine 2B2 hydroxylase, and dopamine transporter expression, for *in vitro* study of PD. GSEA of differentially expressed proteins after overexpression of SIRT4 also supports the possible involvement of SIRT4 in the onset and development of PD. This result is consistent with previous studies of mitochondrial SIRT in neurodegenerative disease (van de Ven et al., 2017).

However, what kind of mechanisms about SIRT4 involved in the onset and development of PD? Previous studies on SIRT4 mainly identified that SIRT4 is an early regulator of branched-chain amino acid catabolism and promotes adipogenesis (Laurent et al., 2013). In non-alcoholic fatty liver disease (NAFLD), SIRT4 deacetylates and destabilizes mitochondrial trifunctional protein-alpha (MTPα), thereby affecting fatty acid β-oxidation (van Maarschalkerweerd et al., 2015). The mTORC1 pathway inhibits cell differentiation and glutamate metabolism by affecting SIRT4 transcription (Csibi et al., 2021). SIRT4 promotes cardiac dysfunction caused by myocardial fibrosis and hypertrophy by increasing ROS levels in response to pathological stimuli (Luo et al., 2017). Our comprehensive analysis of the functional mechanisms of SIRT4 by quantitative proteomic identification and bioinformatics has provided some new perspectives in addition to confirming the changes in the discovered mechanisms. The biological processes identified from an enrichment analysis of differentially expressed proteins after SIRT4 overexpression compared with the CN group included the ribosomal pathway, propionate metabolic pathway, PPAR signalling pathway, fatty acid degradation pathway, and peroxisome pathway. Besides, some other pathways with lower levels of enrichment that deserve attention include circadian rhythm pathway, 5-hydroxytryptaminergic synapses pathway, relaxation signaling pathway, and lipoic acid metabolism pathway. Moreover, the PPI network analysis of differentially expressed proteins mainly enriched in the ribosomal pathway, the PPAR signaling pathway, lipid metabolism pathway, propionate metabolism pathway, phosphatidylinositol metabolism pathway, and glutathione metabolism pathway. What is more important, the most active protein in the PPI network analysis of upregulated proteins was ACOX1, while the most active protein in the PPI network analysis of downregulated proteins was RSL24D1. ACOX1 is associated with the peroxisomal pathway and PPAR signaling pathway, while RSL24D1 is closely related to the ribosomal pathway. Finally, The COG/KOG categories indicated that SIRT4 is most closely associated with translation, ribosome structure, and biogenesis. The multi-level analysis of the above situations shows that SIRT4 functions in dopaminergic neurons mainly by modulating the ribosomal pathway, propionate metabolic pathway, PPAR signaling pathway, and peroxisomal pathway, which compensates to some extent for the lack of previous studies on the mechanisms of SIRT4. Although few studies have examined the roles of SIRT4 in the ribosomal pathway and propionate metabolic pathway, these mechanisms are more or less associated with PD. For example, ribosomal protein s15 is a substrate for LRRK2, a key pathogenic protein in *Drosophila* and human PD models (Martin et al., 2014). Antibodies against ribosomal proteins have been used as predictive markers for age-related neurodegenerative diseases (Yin et al., 2021). In the propionate metabolic pathway, the combined concentrations of acetate and propionate may be a potential biomarker to distinguish MSA patients from PD patients (He et al., 2021). Increasing the level of gut microbiota-derived propionate effectively ameliorated the loss of dopaminergic neurons and motor deficits in a mouse 6-hydroxydopa-induced PD model (Hou et al., 2021). Reduced propionate levels in fecal samples might exert beneficial effects on intestinal epithelial barrier function and motor behavior *via* the AKT signaling pathway in a mouse model of MPTP-induced PD (Huang et al., 2021). In summary, these mechanisms provide new insights into our studies on the function of SIRT4 in PD.

Further confirmation of the regulatory targets of SIRT4 in PD models was achieved by selecting differentially expressed proteins exhibiting higher activity in protein-protein interaction networks as well as differentially expressed proteins with significant changes in major enriched pathways as candidate targets for multilevel screening and validation. Our study revealed only the expression of FABP4 in the PPAR signaling pathway was significantly altered accordingly with increased or decreased SIRT4 expression in SH-SY5Y cells/MPP^+^-treated SH-SY5Y cells and normal mice/MPTP-induced PD mice. Similar to other members of the nuclear receptor superfamily, PPAR is proposed to be a ligand-activated transcription factor involved in lipid, glucose, and amino acid metabolism that simultaneously reprograms immune responses, stimulates metabolic and mitochondrial functions, promotes axonal growth, induces progenitor cell differentiation into myelinated oligodendrocytes, and increases brain clearance of toxic molecules such as β-amyloid (Lautrup et al., 2019). The PPARg agonist pioglitazone has been shown to attenuate MPTP-induced dopamine depletion in a mouse model of PD (Quinn et al., 2008). The beneficial effects of regular treadmill exercise on reducing α-synuclein levels in the brain have been suggested to be mediated by PPARα (Dutta et al., 2022). All of these findings suggest the clinical importance of the PPAR signaling pathway in the field of PD. Maintenance of neuronal metabolic integrity requires moderate lipid transfer between neurons and glial cells via lipid transport proteins. Lipid accumulation in glial cells due to reactive oxygen species production and mitochondrial damage leads to further neuronal damage and degeneration (Ioannou et al., 2019). Moderate cholesterol homeostasis maintains the structure of α-synuclein; however, excess cholesterol promotes the local deposition of α-synuclein and the formation of Lewy vesicles with aggregated α-synuclein, which in turn affects the function of DATs and dopaminergic neurons (van Maarschalkerweerd et al., 2015; Zhuge et al., 2019). Furthermore, the cholesterol metabolite 27-OHC decreases TH activity and causes oxidative stress and apoptosis (Dai et al., 2021). Thus, dyslipidemia is closely associated with PD and LBD, and lipids and small molecules in sebum have even been suggested as potential biomarkers of PD (Klemann et al., 2017; Sinclair et al., 2021). That is to say, abnormal lipid metabolism is clearly an important mechanism in PD. FABP4 is a lipid carrier that is considered a key mediator of systemic metabolic and inflammatory processes (Ron et al., 2021) and is closely associated with the development of metabolic syndrome, inflammation, and atherosclerosis. Importantly, it regulates the onset and development of inflammation and metabolism, resists the progression of atherosclerosis, and improves insulin sensitivity (Furuhashi et al., 2007; Prentice et al., 2021). In other words, FABP4 is a signaling molecule for metabolic abnormalities, and its effects on metabolism, particularly blood glucose and lipid metabolism, will inevitably affect the incidence of PD. Recent studies have also shown that FABP3, belonging to the FABP family, is expressed at high levels in the brain and accelerates α-Syn oligomerization upon cellular exposure to MPTP (Cheng et al., 2019). This finding more or less supports the relevance of FABP4 to PD. SIRT4 plays an important role in lipid metabolism. In skeletal muscle and white adipose tissue, SIRT4 deacetylates and inhibits malonyl coenzyme A decarboxylase (MCD) to regulate lipid metabolism (Laurent et al., 2013). This finding supports the association of SIRT4 with FABP4 to some extent. Although the relationship between the SIRT family and FABP4 has not been explored in PD, upregulation of FABP4 expression in the macrophages of wounds may promote inflammation by decreasing SIRT3 expression in diabetic mice (Boniakowski et al., 2019). FABP4-Cre-mediated SIRT6 deficiency impairs adipose tissue function and metabolic homeostasis in mice (Xiong et al., 2017). These results also confirm that SIRT4, a member of the SIRT family, may be associated with FABP4.

Fatty acid binding proteins are cytoplasmic lipid chaperones that promote fatty acid solubilization, transport, and metabolism and interact with various membrane and intracellular proteins [for example, PPAR, hormone-sensitive lipase (HSL), etc.] (Li et al., 2020). A bidirectional interaction between the FABP4 and PPARγ pathways has been identified to potentially drive the aggressive behavior of tumor cells in bone (Herroon et al., 2013). FABP4 also triggers the ubiquitination and subsequent proteasomal degradation of PPARγ (Herroon et al., 2013). Furthermore, FABP4 induces IL-4-stimulated adipogenesis in human skeletal muscle cells through the activation of the PPARγ signaling pathway (Wang et al., 2022). The correlation between FABP4 and PPARγ is also supported by our findings. However, in the MPTP-induced PD mouse model, SIRT4 overexpression inhibited FABP4 transcription but promoted FABP4 protein expression. This inconsistency between the results obtained at the mRNA and protein levels involves complex mechanisms. Protein abundance depends on the following factors: the transcription rate, mRNA half-life, translation rate [the composition of mRNA sequence codon, upstream open reading frame, internal ribosome entry site (IRES), proteins binding to regulatory elements on transcripts, relative availability of transcript and ribosomes], protein half-life (complex ubiquitin–proteasome pathways or autophagy may affect the protein concentration independent of the transcript concentration), delays in protein synthesis, and protein transport (the protein spatial output separates proteins from synthesized transcripts) (Liu et al., 2016). Among them, the transcription rate and mRNA half-life determine the level of mRNA. Posttranscriptional and posttranslational regulation induce important functional changes in protein abundance, which are not observed at the mRNA level (Schwanhäusser et al., 2011; Kristensen et al., 2013; Baum et al., 2019; Buccitelli and Selbach, 2020). In summary, translation itself is a complex multistep process, and post-translational modifications and protein interactions with other biomolecules together contribute to a phenotype that is more functionally stable than mRNA (Mann and Jensen, 2003; Ryan et al., 2013; Buccitelli and Selbach, 2020). There is no doubt that our results are not contradictory, providing a deeper understanding of the mechanism of SIRT4; however, the exact molecular mechanism requires further elucidation.

Our study, of course, has some limitations. Firstly, although *in vitro* studies exclude the effects of other cell types of the brain on dopaminergic neuronal mechanisms *in vivo*, artificial interference with SIRT4 expression through viral transfection makes the findings different from the real physiological and pathological state, and thus models closer to the disease state are required for verification and confirmation. Secondly, although SH-SY5Y cells with MPP^+^ intervention and MPTP-induced PD mouse model are classical models used in the study of PD, they mainly reflect Parkinson’s disease induced by drug toxicity and do not fully mimic the pathogenic mechanisms of PD. Finally, as the changes in protein are related to differences in transcription, translation, and post-translational modifications, the results of which may vary substantially. Moreover, because changes in protein are related to differences in transcription, translation, and posttranslational modifications, the results may vary substantially. Therefore, multiomics analyses are needed in the future. In conclusion, this study confirms that SIRT4 plays a role in PD models, provides a relatively comprehensive understanding of the possible mechanisms of SIRT4, and identifies the most promising regulatory targets. Although most of the data obtained in this study are still descriptive, our findings provide powerful evidence to support the study of SIRT4-centred biomarkers in PD.

## Data availability statement

The original contributions presented in this study are included in the article/Supplementary material, further inquiries can be directed to the corresponding authors.

## Ethics statement

The animal study was reviewed and approved by the Institutional Committee for Animal Care and Use of Fujian Medical University.

## Author contributions

HW, WS, and KF performed the experiments and wrote the manuscript. GC and YG revised the manuscript. XC, HZ, and QY guided the process and interpreted the results. All authors acquired the data, analyzed the results, read, and approved the final manuscript.

## Supplementary material

Supplementary Figure 1 The first 30 upregulated proteins with significant difference after sirtuin 4 (SIRT4) overexpression in SH-SY5Y cells. The table clearly shows the protein name, molecular weight and G4/CN ratio of the first 30 upregulated proteins. The heat map visually shows the changes of the first 30 upregulated proteins compaired with the control group.

Supplementary Figure 2 The first 30 downregulated proteins with significant difference after sirtuin 4 (SIRT4) overexpression in SH-SY5Y cells. The table clearly shows the protein name, molecular weight and G4/CN ratio of the first 30 downregulated proteins. The heat map visually shows the changes of the first 30 downregulated proteins compaired with the control group.

Supplementary Table 1 Primer sequences for human or mouse RT-PCR experiments.

Supplementary Table 2 Primer sequence and PCR conditions for identification of DJ-1 KO rats.

Supplementary Table 3 The main normalized enrichment score (NES) of differentially expressed proteins after SIRT4 overexpression in SH-SY5Y cells with gene set enrichment analysis.
