# Corrigendum: Proteomic profiling reveals the potential mechanisms and regulatory targets of sirtuin 4 in 1-methyl-4-phenyl-1,2,3,6-tetrahydropyridine-induced Parkinson's mouse model

**DOI:** 10.3389/fnins.2023.1201109

**Published:** 2023-11-23

**Authors:** Huidan Weng, Wenjing Song, Kangyue Fu, Yunqian Guan, Guoen Cai, En Huang, Xiaochun Chen, Haiqiang Zou, Qinyong Ye

**Affiliations:** ^1^Department of Neurology, Fujian Medical University Union Hospital, Fuzhou, China; ^2^Fujian Key Laboratory of Molecular Neurology, Institute of Neuroscience, Fujian Medical University, Fuzhou, China; ^3^Cell Therapy Center, Xuanwu Hospital, Capital Medical University, Beijing, China; ^4^The School of Basic Medical Sciences, Fujian Key Laboratory of Brain Aging and Neurodegenerative Diseases, Fujian Medical University, Fuzhou, China; ^5^Department of Neurosurgery, General Hospital of Southern Theatre Command, PLA, Guangzhou, Guangdong, China

**Keywords:** Parkinson's disease, SIRT4, quantitative proteomic analysis, bioinformatics, biomarkers, FABP4, PPAR signaling pathway

In the published article, an error occurred in the representative immunoblot plot of TH in Figure 2B and Figure 8. The corrected Figure 2 and Figure 8 appear below.

**Figure 2:**
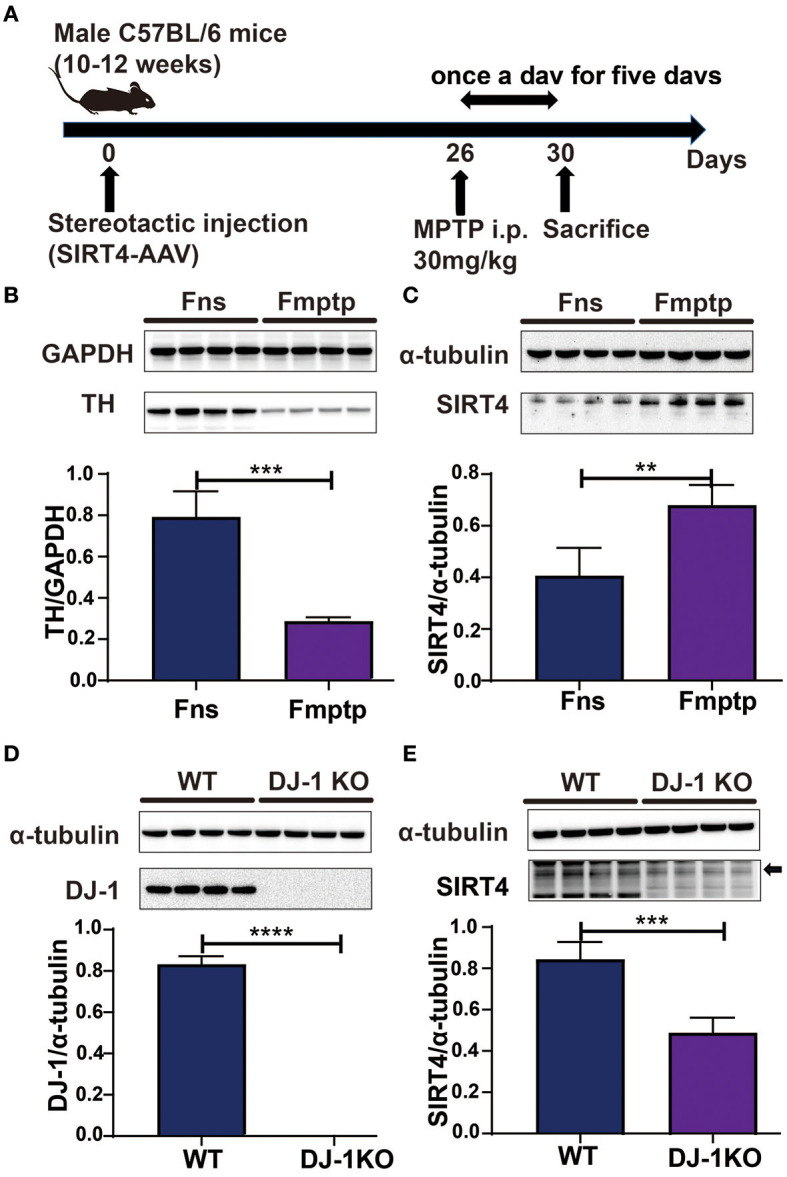
SIRT4 plays a role in a PD model. **(A)** Flow chart of stereotactic injection of SIRT4-AAV virus and the establishment of a mouse model of PD induced by MPTP. **(B)** Representative western blots and densitometric analysis for TH protein between groups. TH protein extracts from substantia nigra after mice were intraperitoneally injected with saline and MPTP for 5 days. GAPDH was used as a loading control (*n* = 4 mice per group). **(C)** Representative western blots and densitometric analysis for SIRT4 protein between groups. SIRT4 protein extracts from substantia nigra after mice were intraperitoneally injected with saline or MPTP for 5 days. Tubulin was used as a loading control (*n* = 4 mice per group). **(D, E)** Representative western blots and densitometric analysis for DJ-1 or SIRT4 protein between groups, respectively. DJ-1 or SIRT4 protein extracts from substantia nigra of WT rats or DJ-1 KO rats. Tubulin was used as a loading control (*n* = 4 mice per group). All results are depicted as means ± SEM. The comparison across groups was analyzed by *t*-test. ^**^P < 0.01, ^***^P < 0.001, and ^****^P < 0.0001 compared with the control group. TH, tyrosine hydroxylase; SIRT4, NAD-dependent protein lipoamidase sirtuin-4, mitochondrial.

**Figure 8:**
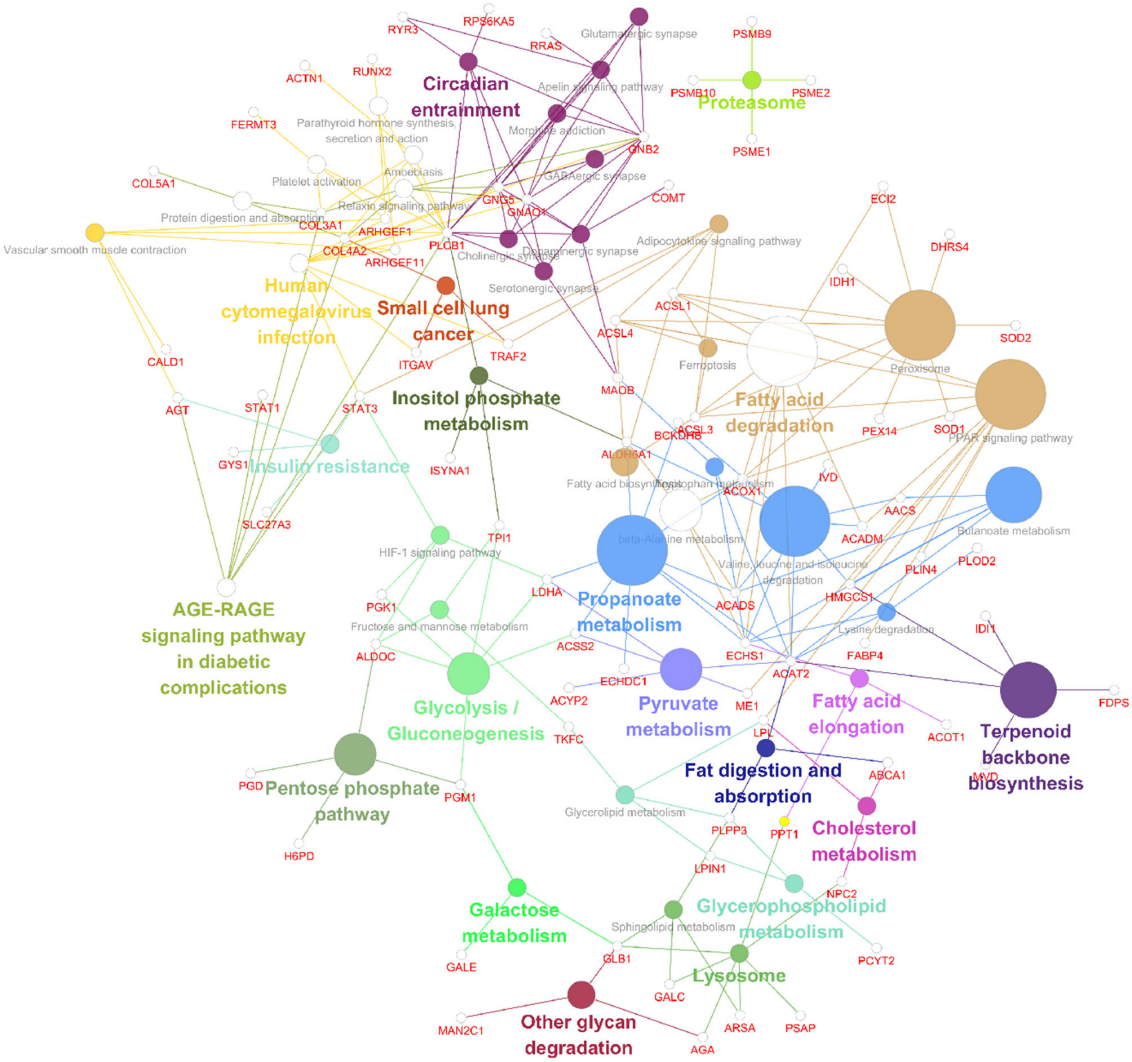
Data visualizations of the results from the KEGG enrichment analysis of differentially expressed genes and proteins in SH-SY5Y cells after SIRT4 overexpression with ClueGO. A KEGG enrichment analysis of differentially expressed proteins in SH-SY5Y cells after SIRT4 overexpression was performed using ClueGO. The figure clearly shows the main proteins in the enriched pathways and the connection between A protein and different pathways. The size of the circle represents the number of different proteins.

The authors apologize for this error and state that this does not change the scientific conclusions of the article in any way. The original article has been updated.

