# Supplementary figures and images for: Proteomic profiling reveals the potential mechanisms and regulatory targets of sirtuin 4 in 1-methyl-4-phenyl-1,2,3,6-tetrahydropyridine-induced Parkinson’s mouse model
Source: Front Neurosci. 2023 Jan 25;16:1035444. doi: 10.3389/fnins.2022.1035444 (PMC9905825; doi:10.3389/fnins.2022.1035444)

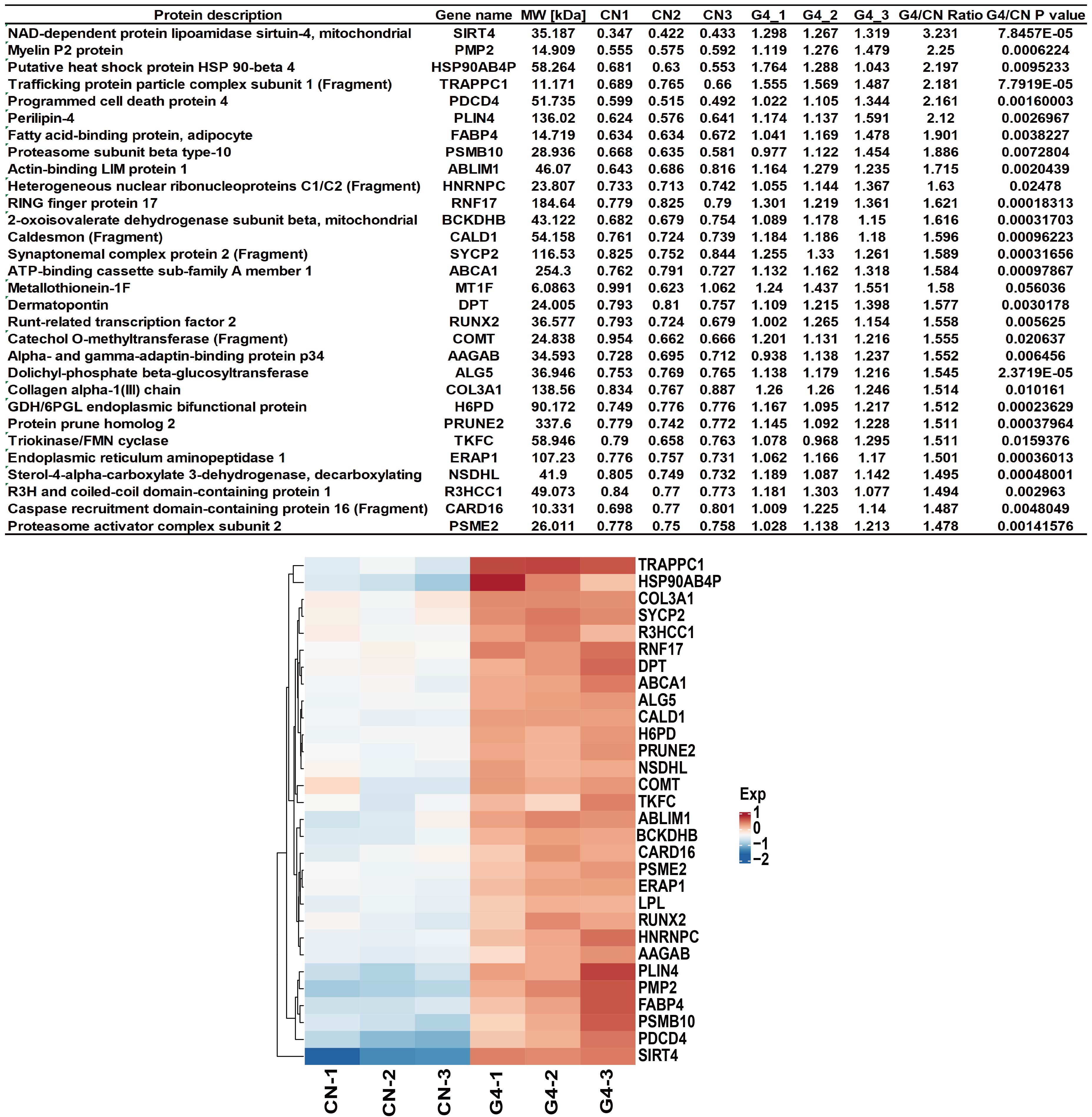

Supplement: Supplementary Figure 1 — The first 30 upregulated proteins with significant difference after sirtuin 4 (SIRT4) overexpression in SH-SY5Y cells. The table clearly shows the protein name, molecular weight and G4/CN ratio of the first 30 upregulated proteins. The heat map visually shows the changes of the first 30 upregulated proteins compaired with the control group. [file Image_1.jpg]

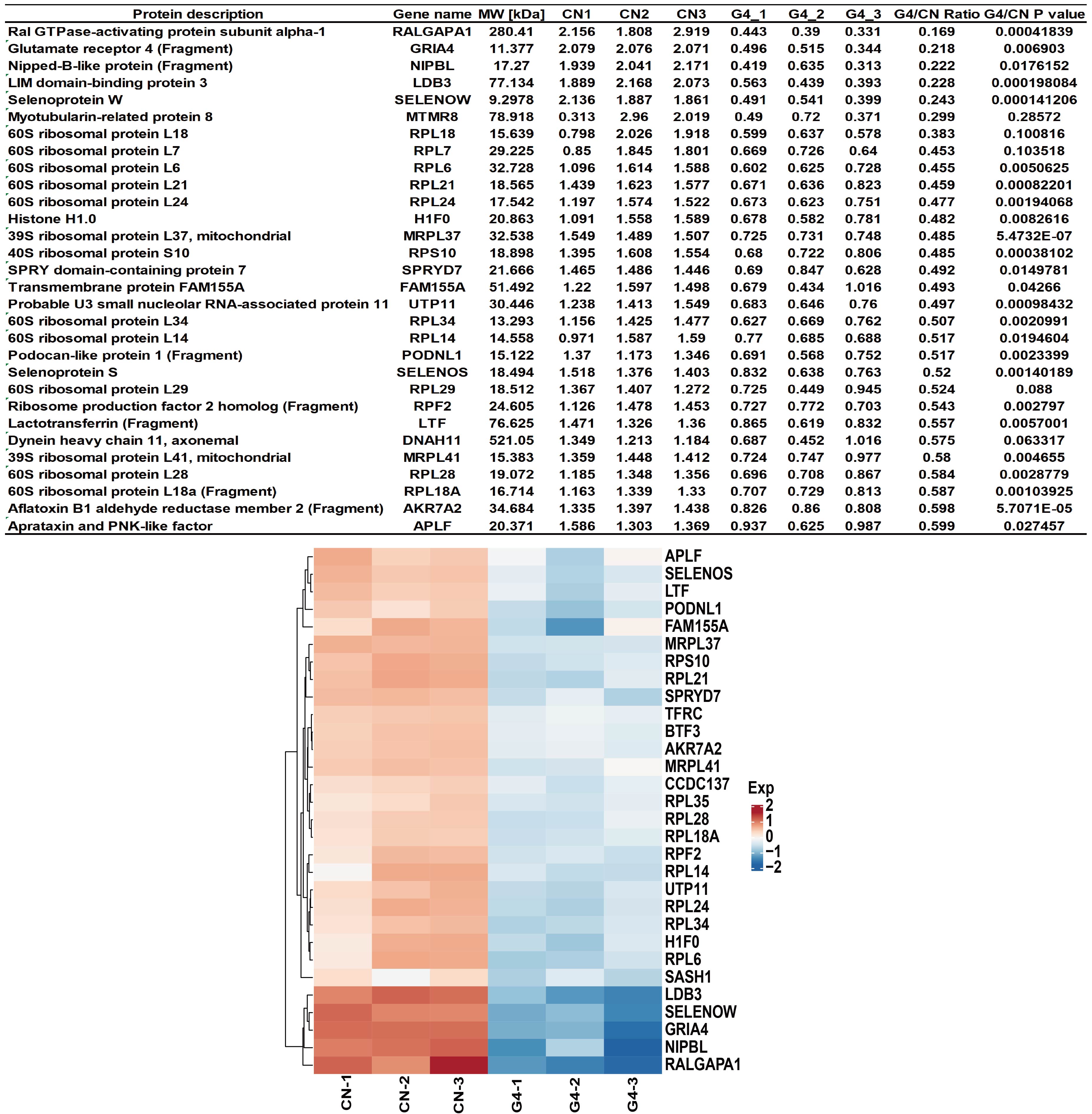

Supplement: Supplementary Figure 2 — The first 30 downregulated proteins with significant difference after sirtuin 4 (SIRT4) overexpression in SH-SY5Y cells. The table clearly shows the protein name, molecular weight and G4/CN ratio of the first 30 downregulated proteins. The heat map visually shows the changes of the first 30 downregulated proteins compaired with the control group. [file Image_2.jpg]
